# The use of frozen plasma samples in thromboelastometry

**DOI:** 10.1007/s10238-017-0454-5

**Published:** 2017-02-16

**Authors:** Christian Schoergenhofer, Nina Buchtele, Michael Schwameis, Johann Bartko, Bernd Jilma, Petra Jilma-Stohlawetz

**Affiliations:** 1 Department of Clinical Pharmacology, Medical University of Vienna, Währinger Gürtel 18-20, 1090 Vienna, Austria; 2 Department of Laboratory Medicine, Medical University of Vienna, Vienna, Austria

**Keywords:** Thromboelastometry, Hyperfibrinolysis, Coagulation, Blood, Blood plasma, Direct oral anticoagulants, Coagulation

## Abstract

Thromboelastometry is increasingly used in the clinical and scientific setting. The use of frozen plasma samples may be useful in overcoming certain limitations such as local and timely availability. Whole blood (WB) samples of 20 healthy volunteers were obtained, and plasma was generated. NATEM (*n* = 20), EXTEM (*n* = 20) and INTEM (*n* = 8) analyses were performed in WB, fresh plasma and frozen and thawed plasma. Dabigatran (500, 1000 ng/ml), rivaroxaban (100, 200 ng/ml) or alteplase (333 ng/ml) were added ex vivo to WB, and thromboelastometry was performed in WB and in frozen and thawed plasma samples. Clot formation time, mean clot firmness and the area under the curve were significantly altered in plasma compared to WB. In INTEM and EXTEM analysis, clotting time (CT) was comparable between WB (100%) and fresh (INTEM 114% and EXTEM 93%, ratio of the means) and frozen plasma samples (85 and 99%), whereas in NATEM analysis, the CT increased in fresh (193%) and frozen plasma samples (130%). Dabigatran dose-dependently increased the CT approximately 5- and 9-fold in WB and even more pronounced 10- and 26-fold in plasma. Accordingly, rivaroxaban dose-dependently increased the CT 2- and 2.7-fold in WB, and 3.5- and 4-fold in plasma samples. Hyperfibrinolysis was achieved by addition of alteplase in all WB samples and was reproducible in plasma samples. In conclusion, thromboelastometry, especially INTEM and EXTEM analyses, is possible using frozen and stored plasma samples with comparable results to the corresponding whole blood samples.

## Introduction

Thromboelastometry is a point-of-care assay measuring the viscoelastic properties of clot formation and clot lysis of whole blood [1]. The results of such measurements offer quick information about the global coagulation profile [2].

This may be useful in certain clinical situations, i.e., during surgery, in bleeding patients, in patients with hemorrhagic disorders, to monitor certain drugs or when massive transfusions are required [3–9]. Furthermore, the impact of hemostatic interventions can be rapidly assessed and decisions may be based on results of such viscoelastic tests [10]. The advantages of thromboelastometry-guided hemostatic therapy were demonstrated in patients undergoing cardiac surgery [11]. Blood loss and the need for fresh frozen plasma units could be reduced. Moreover, the perioperative need of blood products could be reduced in patients with severe burn injuries [12]. The diagnosis of hyperfibrinolysis is often difficult and testing the viscoelastic properties of blood may offer important information to support the diagnosis [13, 14] or to diagnose hyperfibrinolytic disseminated intravascular coagulation [15]. Thromboelastometry is also sensitive to anticoagulant drugs such as enoxaparin [16], apixaban, rivaroxaban, argatroban and dabigatran [17–19].

Thromboelastometry is sensitive to the coagulation activation in the human endotoxemia model [20] and may be used to identify prothrombotic states in patients [21–23].

However, like every other testing system some limitations need to be considered. First of all, thromboelastometry produced accurate and reproducible results within 30 min up to 4 h, but inconsistent references are published on the stability and reproducibility of measurements after sample storage at room temperature [24–26]. The intra-individual coefficient of variation of thromboelastometry ranged between 11 and 23% in healthy volunteers (own unpublished data). Secondly, trained personnel and available devices are necessary. In the clinical setting, usually rapid results are required which is an important advantage of this method. On the contrary, in the scientific setting, storage of samples for later batch analysis offers obvious advantages.

The aim of this study was to investigate whether the results of thromboelastometry performed on frozen plasma samples are comparable to the results obtained from freshly obtained whole blood. This may help to overcome at least part of these limitations.

## Methods

This study was performed at the Department of Clinical Pharmacology at the Medical University of Vienna. The independent Ethics Committee of the Medical University of Vienna approved the study. Twenty healthy volunteers were included in the study, and blood sampling was performed between February and April 2016 (Table 1).

**Table 1:** Demographics and baseline data of participants

Parameters	Mean ± SD
Gender m (f)	11 (9)
Age (years)	26 ± 5
Height (cm)	174 ± 10
Weight (kg)	72 ± 17
Hemoglobin (g/dl)	14.1 ± 1.3
Platelets (*10^^9^)	265 ± 59
Leukocytes (*10^^9^)	5.9 ± 1.3

Baseline data and demographics are presented (means ± SD)

### Preparation of whole blood and plasma samples

Whole blood samples were obtained from fresh venipunctures in healthy volunteers and collected into 3.8% sodium citrate tubes.

To generate platelet-free plasma, whole blood was centrifuged twice at 2500*g* for 15 min at room temperature and the supernatant was transferred into polypropylene tubes. Plasma samples were either used for immediate analysis (fresh plasma) or frozen and stored as explained below (frozen plasma).

To investigate the effects of freezing on hyperfibrinolysis, we spiked alteplase (actilyse, 333 ng/ml) into whole blood samples to induce artificial hyperfibrinolysis. ROTEM analysis was performed for fresh blood, and again plasma was generated for immediate (fresh plasma) or later (frozen plasma) analysis as explained above.

To investigate the effects of oral anticoagulants, we spiked rivaroxaban (100 and 200 ng/ml) and dabigatran (500 and 1000 ng/ml) into whole blood and performed ROTEM analysis. Again plasma for immediate and later analysis was generated from the whole blood samples as explained above.

Plasma samples of all 20 healthy volunteers were frozen and stored at −80 °C until later analysis. These samples included native plasma and plasma containing rivaroxaban, dabigatran and alteplase, as explained above.

Additionally, we investigated whether different freezing techniques and storage temperatures affected the results of ROTEM analysis. Therefore, plasma of eight healthy volunteers was frozen using dry ice (−78 °C) and then transferred to −80 or −18 °C storage. Plasma samples of another eight subjects were frozen and stored at −18 °C, and plasma samples of another eight subjects were frozen using liquid nitrogen (−196 °C) and then transferred to −80 or −18 °C storage.

All frozen plasma samples were thawed using a water bath heated to 37 °C (C) for 15 min and analyzed thereafter. Whole blood and fresh plasma samples were tested at the same time, whereas frozen plasma samples were analyzed at one to four weeks later.

### Thromboelastometry

The viscoelastic properties of whole blood or plasma were investigated with the ROTEM coagulation analyzer (Pentapharm, Munich, Germany) as previously described [20]. In short, ROTEM measures shear elastic modulus during clot formation and subsequent fibrinolysis. The ROTEM uses a ball-bearing system for power transduction, which makes it less susceptible to mechanical stress, movement and vibration.

ROTEM measurements produced accurate and reproducible results within 30 min up to 4 h. In fresh blood samples, we performed ROTEM measurements between 1 and 2 h after blood storage at room temperature.

Just before running the assay, citrated blood samples were recalcified with 20 µl of CaCl_2_ 0.2 M (Start-TEM; Nobis, Endingen, Germany) and the test was started. We performed thromboelastometry without adding additional activators (NATEM), as well as EXTEM analysis (recombinant tissue factor- and phospholipid-activated ROTEM), and in eight subjects INTEM analysis (partial thromboplastin–phospholipid-activated ROTEM). The following ROTEM parameters were analyzed: the clotting time (CT), the clot formation time (CFT), the maximum clot firmness (MCF), the alpha angle (alpha), the maximum lysis (ML), the time of maximum lysis (LT) and the area under the curve (AUC).

In samples with alteplase, dabigatran or rivaroxaban, only EXTEM analysis was performed.

ROTEM analysis in frozen plasma samples was generally technically feasible. However, one problem regularly occurred in the testing system and an error code was reported that the stability of the clot rapidly increased and the sample dried. All tests were running for 2 h. This problem may have affected the detection of ML and the AUC, because these parameters are measured continuously until the end of the chosen running time.

Of note, the clot signal amplitude did not reach 20 mm in all plasma samples resulting in no measurable CFT.

### Statistical analysis

A formal sample size calculation was not performed, because no data on this topic were available. We performed nonparametric testing using the Friedman ANOVA for overall comparisons and the Mann–Whitney-*U* test for group-wise comparisons. Descriptive statistics are presented as means and standard deviations unless otherwise stated.

## Results

### ROTEM results

Table 2 presents the results of ROTEM analysis performed in whole blood samples, fresh and frozen plasma samples.

**Table 2:** Results of ROTEM performed in whole blood, plasma and plasma frozen and stored at −80 °C

Para-meters	NATEM (*n* = 20)			INTEM (*n* = 8)			EXTEM (*n* = 20)		
	Whole blood	Fresh plasma	Frozen plasma	Whole blood	Fresh plasma	Frozen plasma	Whole blood	Fresh plasma	Frozen plasma
CT (s) (mean ± SD % of whole blood)	856 ± 233	1649 ± 659** (289 ± 220%)	1113 ± 313**^$^ (81 ± 26%)	226 ± 54	259 ± 60^$^ (163 ± 93%)	191 ± 35 (91 ± 53%)	67 ± 8	62 ± 9 (83 ± 33%)	66 ± 11 (105 ± 20%)
CFT (s)	357 ± 194	861 ± 894**^a^ (228 ± 84%)	1054 ± 1143**^a^ (49 ± 37%)	86 ± 15	766 ± 802**^a^ (463 ± 648%)	1321 ± 2888**^a^ (56 ± 45%)	91 ± 20	735 ± 1217**^a^ (497 ± 847%)	816 ± 858**^a^ (26 ± 26%)
Alpha angle (°)	44 ± 10	28 ± 12** (60 ± 29%)	28 ± 12** (153 ± 64%)	73 ± 3	72 ± 5^$^ (78 ± 31%)	78 ± 3* (103 ± 53%)	72 ± 4	79 ± 2** (99 ± 35%)	79 ± 2** (91 ± 4%)
MCF (mm)	53 ± 6	27 ± 10** (52 ± 26%)	25 ± 5** (219 ± 32%)	63 ± 4	26 ± 14 (44 ± 15%)	23 ± 5** (225 ± 110%)	64 ± 5	26 ± 9** (38 ± 18%)	26 ± 10** (271 ± 62%)
ML (%)	13 ± 3	n.a.	n.a.	14 ± 4	n.a.	n.a.	18 ± 3	n.a.	n.a.
AUC	4986 ± 1350	2609 ± 1140** (50 ± 48%)	2503 ± 556** (214 ± 34%)	5376 ± 2364	2604 ± 877** (49 ± 20%)	2294 ± 506** (223 ± 110%)	6358 ± 448	2550 ± 1112** (36 ± 20%)	2539 ± 1066** (282 ± 93%)

Results of ROTEM analysis (NATEM, INTEM and EXTEM) performed in whole blood, plasma and plasma frozen and stored at −80 °C (mean ± SD). Statistical testing was performed between whole blood and plasma and plasma frozen and stored at −80 °C (*n* = 20)

Mean ± SDs are presented. *n.a.* not available, *CT* clotting time, *CFT* clot formation time, *MCF* mean clot firmness, *Alpha* alpha angle, *ML* maximum lysis, *AUC* area under the curve

* *p* < 0.05, ** *p* < 0.01 versus whole blood samples, ^$^ *p* < 0.05 plasma versus −80 °C

^a^A clot signal amplitude of 20 mm was not reached in all samples

### Whole blood versus fresh and frozen plasma

#### NATEM

The CT was approximately 50% shorter in whole blood compared to fresh plasma (*p* < 0.001) and 25% compared to frozen plasma samples (*p* = 0.003). Interestingly, the CT was also shorter in frozen plasma samples compared to fresh plasma (*p* = 0.005). The CFT was approximately two-fold longer in fresh or frozen plasma compared to whole blood (*p* < 0.001). Expectedly, MCF and AUC were approximately two-fold higher in whole blood than in plasma samples due to a lack of blood cells (*p* < 0.001).

#### EXTEM

**Fig. 1:**
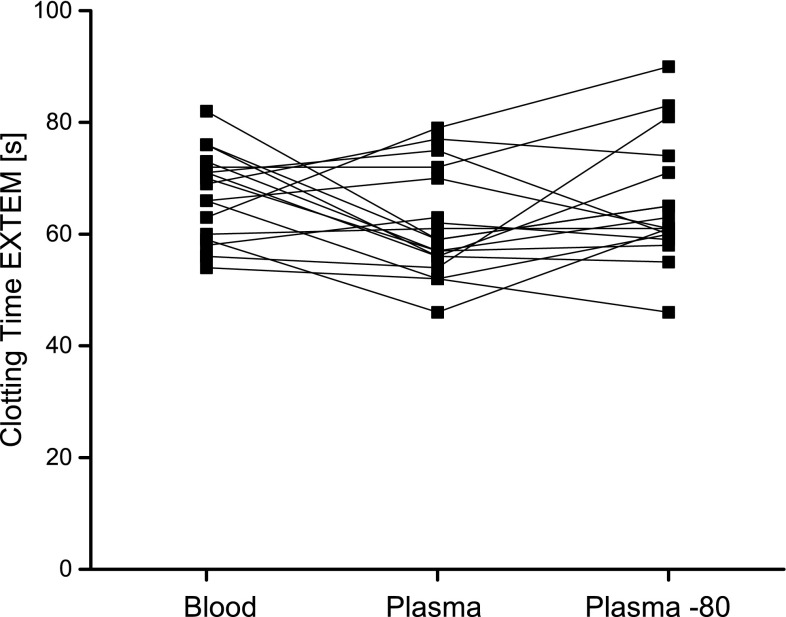
Individual clotting times in EXTEM analysis using whole blood, plasma and frozen and thawed plasma (plasma—stored at −80 °C) (*n* = 20)

The CT was similar between whole blood, fresh and frozen plasma samples. However, a trend to a shorter CT was noticeable in fresh plasma samples (*p* = 0.086). Individual CTs are shown in Fig. 1. The CFT was 8- to 9-fold longer in fresh and frozen plasma samples compared to whole blood, respectively (*p* < 0.001). Alpha angles were approximately 10% higher in plasma samples compared to whole blood (*p* < 0.001). Expectedly, the AUC and the MCF were approximately 2.5-fold higher in whole blood compared to fresh or frozen plasma samples (*p* < 0.001). Similar to NATEM analysis, measurement of ML was not feasible in plasma samples.

#### INTEM

The CT was similar between whole blood and fresh or frozen plasma samples. However, the CT in frozen plasma was shorter than in fresh plasma samples (*p* = 0.006). The CFT was 9- to 15-fold longer in fresh and frozen plasma samples compared to whole blood, respectively (*p* = 0.01 and *p* = 0.014). The alpha angle was 10% smaller in whole blood and fresh plasma samples compared to frozen plasma samples (*p* = 0.008 and *p* = 0.042, respectively). Similar to NATEM and EXTEM analysis, the MCF (*p* ≤ 0.001) and the AUC (*p* < 0.03) were approximately 2.5-fold higher in whole blood compared to plasma samples.

### Freezing methods

#### NATEM

The CT was similar for whole blood and plasma frozen and stored at −18 °C and for plasma frozen in dry ice and stored at −80 °C, but significantly longer for plasma frozen and stored at −80 °C (*p* = 0.003), frozen with dry ice and stored at −18 °C (*p* = 0.003), frozen with liquid nitrogen and stored at −18 °C (*p* = 0.004) and frozen with liquid nitrogen and stored at −80 °C (*p* = 0.001) (Table 3). The CFT was significantly longer in plasma samples compared to whole blood, except for samples frozen and stored at −18 °C. Expectedly, the AUC, MCF and alpha angles were significantly smaller in plasma compared to whole blood, except for the alpha angles measured in samples frozen and stored at −18 °C. Again, ML was not measureable.

**Table 3:** Results of ROTEM performed in plasma samples frozen by different techniques and stored at different temperatures

	Whole blood (*n* = 20)	−18 °C (*n* = 8)	−80 °C (*n* = 20)	Dry ice −18 °C (*n* = 8)	Dry ice −80 °C (*n* = 8)	Fluid N_2_ −18 °C (*n* = 8)	Fluid N_2_ −80 °C (*n* = 8)
*NATEM*							
CT (s) (mean ± SD % of whole blood)	856 ± 233	932 ± 172 (130 ± 41%)	1113 ± 313** (81 ± 26%)	1200 ± 285** (167 ± 45%)	979 ± 210 (151 ± 60%)	1256 ± 299** (184 ± 85%)	1402 ± 501** (141 ± 70%)
CFT (s)	357 ± 195	937 ± 1210 (320 ± 367%)	1054 ± 1143^$^ (49 ± 37%)	654 ± 326** (246 ± 129%)	792 ± 513** (274 ± 228%)	1266 ± 1482** (213 ± 120%)	884 ± 495** (287 ± 230%)
Alpha angle (°)	44 ± 10	35 ± 12 (70 ± 16%)	28 ± 12^$^ (153 ± 64%)	32 ± 5** (63 ± 10%)	31 ± 12* (68 ± 30%)	28 ± 9** (68 ± 11%)	27 ± 11** (68 ± 21%)
MCF (mm)	53 ± 6	23 ± 9^$^ (42 ± 16%)	25 ± 5^$^ (219 ± 32%)	24 ± 2^$^ (44 ± 4%)	20 ± 7^$^ (38 ± 14%)	23 ± 4^$^ (46 ± 8%)	22 ± 3^$^ (40 ± 7%)
AUC	5310 ± 669	2352 ± 998^$^ (42 ± 16%)	2503 ± 556^$^ (214 ± 34%)	2114 ± 937^$^ (39 ± 18%)	2060 ± 928^$^ (38 ± 17%)	2321 ± 424^$^ (46 ± 7%)	2294 ± 266^$^ (42 ± 6%)
*EXTEM*							
CT (s)	67 ± 8	57 ± 10* (92 ± 25%)	66 ± 11 (105 ± 20%)	60 ± 10 (97 ± 21%)	62 ± 7 (98 ± 7%)	66 ± 12 (107 ± 24%)	76 ± 37 (119% ± 52%)
CFT (s)	91 ± 20	885 ± 1273* (1115 ± 1688%)	816 ± 858^$^ (26 ± 26%)	1263 ± 2010^$^ (1355 ± 1918%)	499 ± 317^$^ (583 ± 277%)	2356 ± 2632^$^ (1626 ± 1978%)	880 ± 1097^$^ (857 ± 911%)
Alpha angle (°)	72 ± 4	78 ± 5** (106 ± 5%)	79 ± 2^$^ (91 ± 4%)	80 ± 1^$^ (92 ± 41%)	79 ± 1^$^ (107 ± 3%)	78 ± 2^$^ (109 ± 4%)	76 ± 6* (106 ± 11%)
MCF (mm)	64 ± 5	23 ± 6^$^ (34 ± 8%)	26 ± 10^$^ (271 ± 62%)	23 ± 3^$^ (35 ± 3%)	22 ± 3^$^ (34 ± 3%)	21 ± 3^$^ (33 ± 4%)	23 ± 4^$^ (36 ± 7%)
AUC	6358 ± 448	2271 ± 638^$^ (34 ± 8%)	2539 ± 1066^$^ (282 ± 93%)	1915 ± 852^$^ (29 ± 13%)	1881 ± 831^$^ (28 ± 13%)	2054 ± 303^$^ (32 ± 4%)	2295 ± 372^$^ (35 ± 6%)

This table presents results (mean ± SD) of NATEM and EXTEM analysis of plasma samples frozen and stored at −18 °C, frozen with dry ice and stored at −18 °C, frozen with dry ice and stored at −80 °C, frozen with liquid nitrogen and stored at −18 °C, frozen with liquid nitrogen and stored at −80 °C

*CT* clotting time, *CFT* clot formation time, *MCF* maximum clot firmness, *Alpha* alpha angle, *AUC* area under the curve

* *p* < 0.05, ** *p* < 0.01, ^$^ *p* < 0.001

#### EXTEM

The CT was shorter in samples frozen and stored at −18 °C compared to whole blood (*p* = 0.025), but similar for all other freezing and storage methods. The CFT was significantly longer in all plasma samples, the AUC and the MCF were significantly smaller compared to whole blood. Alpha angles were approximately 10% larger in all plasma samples compared to whole blood.

### Dabigatran and rivaroxaban anticoagulation

**Fig. 2:**
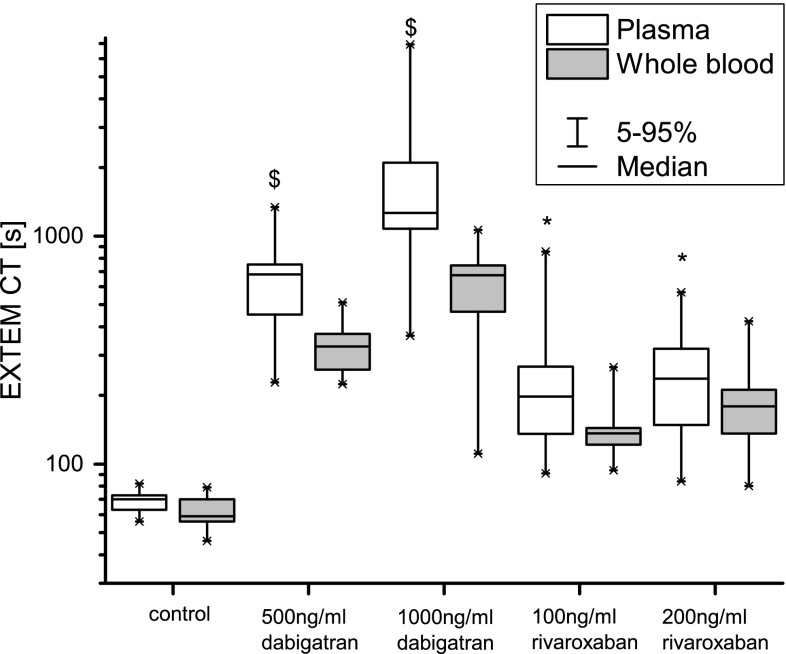
Clotting times after addition of dabigatran and rivaroxaban to whole blood and plasma. Whole blood was spiked in vitro with two doses of dabigatran (500 and 1000 ng/ml) or rivaroxaban (100 and 200 ng/ml). After EXTEM analysis was performed in whole blood, the sample was centrifuged, frozen and stored at −80 °C. Frozen plasma samples were thawed, and EXTEM analysis was performed. *Boxplots* present medians, 25–75% quartiles and 5–95% percentiles

Expectedly, ex vivo addition of dabigatran and rivaroxaban to whole blood prolonged the CT significantly and concentration-dependently in whole blood and in plasma samples (Fig. 2). The CT and CFT of plasma samples were significantly longer for plasma samples compared to whole blood samples (Fig. 2, *p* < 0.001 for both dabigatran, rivaroxaban concentrations: *p* = 0.019 for 100 ng/mL, *p* = 0.021 for 200 ng/mL). The MCF and the AUC were significantly smaller in plasma samples compared to whole blood (*p* < 0.001 for all tests). The alpha angle was larger in plasma samples compared to whole blood (*p* < 0.001).

### Hyperfibrinolysis

Almost complete clot lysis occurred in whole blood samples, which was well reproducible in plasma samples (Table 4). Whole blood and plasma samples differed for CFT, MCF and the AUC. However, CT, ML and LT were similar.

**Table 4:** Results of EXTEM analysis after addition of alteplase

	Whole blood samples (*n* = 10)	Plasma −80 (*n* = 10)	Individual % of whole blood (mean ± SD)	*p* value
CT (s)	69 ± 9	64 ± 9	109 ± 17%	n.s.
CFT (s)	91 ± 23	422 ± 270^a^	40 ± 47%	0.001
MCF (mm)	52 ± 6	19 ± 5*	300 ± 91%	<0.001
Alpha angle (°)	72 ± 4	72 ± 25*	92 ± 4%	0.003
ML (%)	100 ± 0	99 ± 3	101 ± 3%	n.s.
AUC	5188 ± 605	1822 ± 453*	301 ± 85%	<0.001
LT (s)	1655 ± 1129	1053 ± 340	138 ± 49%	n.s.

This table presents the results (means ± SD) of EXTEM analysis after alteplase (333 ng/ml) was added to whole blood samples. After analysis was performed, plasma was generated, frozen and stored at −80 °C, and thawed for analysis. CT and clot lysis-specific parameters ML and LT were not different between whole blood and plasma samples

*CT* clotting time, *CFT* clot formation time, *MCF m*aximum clot firmness, *Alpha* alpha angle, *ML* maximum lysis, *AUC* area under the curve, *LT* time of maximum lysis,* n.s.* not significant

* *p* < 0.05

^a^A clot signal amplitude of 20 mm was not reached in all samples

## Discussion

We investigated whether ROTEM analysis performed in frozen, stored and thawed plasma samples provides similar results compared to whole blood samples. We only found few reports performing thromboelastometry in human plasma samples [27–30]. Based on these, we expected a smaller MCF, a prolonged CFT and a reduced AUC due to a lack of blood cells. Clotting time was comparable in EXTEM and, to a lesser degree, in INTEM analysis.

In EXTEM analysis, the CT tended to be shorter in plasma samples. This difference, although statistically significant, was minor, and the clinical relevance is at least questionable. Of note, all results were within the reference values suggested by the manufacturer (43–82 s) [31]. Moreover, another study measured a relatively short median CT of 47 s in EXTEM analysis using platelet free plasma [27].

In contrast to EXTEM, INTEM and NATEM analysis showed a prolonged CT in fresh plasma samples compared to whole blood. INTEM analysis uses a less potent activator compared to EXTEM analysis, while NATEM analysis does not involve any activator. This suggests that in the absence of strong activators pro-coagulant surfaces seem to be more important for the initiation of the coagulation cascade. Indeed, in some samples coagulation activation did not occur at all or was prolonged to more than 3000 s. The CT of NATEM analysis in whole blood was longer in our population (856 ± 233 s) compared to the manufacturer’s reference values (254–837 s) [31] and compared to other studies [20, 32]. However, the variability of results in the non-activated thromboelastometry analysis was similar to these studies.

We furthermore investigated various freezing methods in a small number of samples (*n* = 8), and except for samples frozen and stored at −18 °C and the samples frozen in dry ice and stored at −80 °C, the CT in NATEM analysis was 40–60% longer compared to whole blood samples. In EXTEM analysis, no differences were detectable except for the samples frozen and stored at −18 °C, in which the CT was ~10% shorter. It is conceivable that freezing, storage and/or thawing procedures have an impact on the results. One possible explanation for the varying results may be cold or contact activation of coagulation factors, which was shown for factor V, VIII, XII and consecutive thrombin generation [33, 34]. To minimize the influence of such factors, we applied a strict freezing and thawing schedule. We used citrate-anticoagulated blood samples as suggested by the manufacturer. However, in contrast to citrate, corn trypsin inhibitor sufficiently suppressed contact activation in calibrated automated thrombin generation [35–37]. Thus, it is possible that the use of corn trypsin inhibitor could improve the reproducibility of results. Numerically, the shortest CT of all plasma samples was measured in samples frozen and stored at −18 °C. Cold activation may have played the biggest part in these samples, because freezing itself takes longer than with snap-freezing. We therefore cannot recommend freezing and storing samples at −18 °C. Considering our results and given the limited availability of fluid N2 and dry ice, we recommend freezing and storing of plasma samples at −80 °C.

Residual platelet counts affect global coagulation tests or coagulation factors only by a small margin [38]. However, we hypothesized that in the non-activated setting, the presence of pro-coagulatory surfaces may be of greater importance. To obtain platelet-free plasma, we performed two centrifugation steps at 2500*g*. We measured platelet counts in a smaller number of plasma samples and detected a median platelet count <1 G/L. However, we cannot exclude entirely that very low platelet counts occurred in some samples. Thus, disintegration of any residual platelets and consecutive exposure to phospholipids may have led to the shorter clotting time in frozen plasma samples [39].

The influence of dabigatran and rivaroxaban on viscoelastic properties of whole blood was demonstrated previously [17, 40]. In plasma samples, the CT was further increased compared to whole blood. As we used the same whole blood samples for plasma generation as for ROTEM analysis, the concentration itself remained equal. It seems likely that in the presence of coagulation inhibitors such as rivaroxaban or dabigatran, the activator used in EXTEM analysis may not be potent enough to initiate coagulation and the lack of pro-coagulant surfaces becomes relevant. The CT is therefore further prolonged. Due to the magnitude of the observed effects, the use of frozen plasma samples in EXTEM analysis to investigate the presence of inhibitors of coagulation such as rivaroxaban or dabigatran seems especially suitable.

Thromboelastometry may be used as a supportive tool in the diagnosis of hyperfibrinolysis [14]. To artificially induce hyperfibrinolysis, we spiked alteplase into blood samples as described earlier [13, 41]. Hyperfibrinolysis was reproducible in frozen and stored plasma samples with a shorter time of maximum lysis in plasma samples. A likely explanation may be the reduced MCF in plasma samples with quicker complete lysis of the smaller clot. Interestingly, no difference was detected in the CT of plasma samples compared to whole blood indicating the reproducibility of the results. However, due to the mentioned technical limitations caused by the regular clotting of the entire sample, we assume that only hyperfibrinolysis of a certain extent is detectable in plasma samples. As we included only healthy volunteers with minimal clot lysis and a usually late onset of clot lysis in our study, we were not able to detect any form of clot lysis. In hyperfibrinolytic patients, lysis occurs within 30 min with a complete clot lysis within 60 min [14]. This faster onset and the strong fibrinolysis should be initiated and therefore be detectable before the sample has clotted entirely. However, this needs to be confirmed in studies investigating hyperfibrinolysis in patients.

This study has several limitations. First of all, the sample size of 20, and in some instances 8, may be too small to detect differences or may be affected by outliers. Furthermore, we did not perform global or more specific coagulation tests before inclusion in the study or in thawed plasma samples. When ROTEM was performed in plasma samples, the error “dried plasma sample” occurred regularly, which probably affected measurement of low level ML. We only included healthy volunteers in our study. Possibly in thrombophilic conditions, NATEM analysis is more reliable due to the presence of activators of coagulation such as tissue factor.

In conclusion, thromboelastometry may be performed in frozen, stored and thawed plasma samples taking the mentioned limitations into account. Activated ROTEM tests such as INTEM or EXTEM are reproducible, and results are comparable between whole blood and corresponding fresh and frozen plasma, whereas non-activated NATEM analysis is subject to greater variability. Our test setup was suitable to detect potent hyperfibrinolysis with a rapid onset and the presence of anticoagulants.

## List of abbreviations

AUC: Area under the curve
C: Celsius
CT: Clotting time
CFT: Clot formation time
MCF: Maximum clot firmness
ML: Maximum lysis
LT: Time of maximum lysis
